# Combined Effects of Physiotherapy and Vitamin D Supplementation on Pain, Disability, and IL-6 Expression in Chronic Low Back Pain: A Randomized Controlled Trial

**DOI:** 10.3390/life16081288

**Published:** 2026-08-04

**Authors:** Abdulaziz A. Albalwi, Hamad S. Al Amer, Rashid Mir, Shahul Hameed Pakkir Mohamed, Jamsheed Javid, Mohammad Muzaffar Mir, Waad Alamri, Yousef M. Alshehre, Ahmad A. Alharbi

**Affiliations:** 1 Department of Health Rehabilitation Sciences, Faculty of Applied Medical Sciences, University of Tabuk, Tabuk 71491, Saudi Arabia; aa-albalwi@ut.edu.sa (A.A.A.); s-mohamed@ut.edu.sa (S.H.P.M.); yalshehre@ut.edu.sa (Y.M.A.); aaalharbi@ut.edu.sa (A.A.A.); 2 Prince Fahd Bin Sultan Chair for Biomedical Research, University of Tabuk, Tabuk 71491, Saudi Arabia; rashid@ut.edu.sa (R.M.); alamri.waad96@gmail.com (W.A.); 3 Department of Medical Laboratory Technology, Faculty of Applied Medical Sciences, University of Tabuk, Tabuk 71491, Saudi Arabia; jali@ut.edu.sa; 4 Department of Clinical Biochemistry, College of Medicine, University of Bisha, Bisha 61922, Saudi Arabia; mmmir@ub.edu.sa

**Keywords:** low back pain, physical therapy modalities, exercise therapy, electric stimulation therapy, vitamin D, interleukin-6

## Abstract

Background: Chronic low back pain (CLBP) is a leading cause of global disability, linked to elevated IL-6 and vitamin D deficiency or insufficiency. While interferential current (IFC) therapy and exercise therapy are established treatments, their combined effect with vitamin D supplementation on inflammatory biomarkers, pain, and functional disability remains underexplored. Objective: The aim of this study was to evaluate the combined effects of physiotherapy and vitamin D supplementation on pain, disability, IL-6 expression, and vitamin D levels in CLBP patients. Methods: This two-arm, parallel-group, randomized controlled trial enrolled 60 CLBP patients, randomized into Group A (physiotherapy alone: IFC therapy and exercise therapy; *n* = 30) or Group B (physiotherapy plus vitamin D supplementation; *n* = 30). Outcomes included pain intensity (11-point Numeric Pain Rating Scale (NPRS)), disability (Arabic Oswestry Disability Index (ODI), 0–100%), and serum IL-6 and 25-hydroxyvitamin D (both by ELISA; pg/mL and ng/mL, respectively), which were assessed at baseline and 6 weeks. All 60 participants enrolled had laboratory-confirmed vitamin D deficiency or insufficiency (serum 25(OH)D < 20 ng/mL) at screening. Independent samples *t*-tests compared continuous baseline characteristics and between-group differences in 6-week change scores; paired *t*-tests assessed within-group change from baseline to 6 weeks; the chi-square test compared the categorical variable of gender (*α* = 0.05). Results: Both groups showed significant improvements in NPRS and ODI at six weeks (*p* < 0.001). Group B demonstrated significant reductions in NPRS (−3.01 vs. −1.71) and ODI (21.83% vs. 16.33%). IL-6 decreased significantly only in Group B (18.24 pg/mL, *p* < 0.001), with no significant change in Group A (25.96 pg/mL, *p* = 0.626). Vitamin D levels increased significantly in Group B (31.68 ng/mL, *p* < 0.001) compared with Group A (19.68 ng/mL, *p* = 0.115), a between-group difference that was also statistically significant. Conclusions: Combining physiotherapy with vitamin D supplementation improves outcomes in the management of CLBP. This approach effectively reduces pain, functional disability, inflammation, and vitamin D deficiency or insufficiency, offering a comprehensive, evidence-based treatment strategy.

## 1. Introduction

Low back pain (LBP) is one of the leading global causes of years lived with disability, with point prevalence estimates of approximately 8–15% of adults, distinct from the lifetime prevalence of low back pain in general, which has been reported to be as high as 80% [1]. Chronic low back pain (CLBP) is characterized by pain lasting more than 12 weeks and is associated with significant socioeconomic burdens, decreased quality of life, and reduced functional ability [2]. The pathophysiology of CLBP involves complex interactions between mechanical stress, inflammation, and genetic factors [3]. The harmful effects associated with low back pain (LBP) are extensive, emphasizing the need for innovative therapies to prevent and treat this condition and its related symptoms [4]. Emerging evidence suggests that CLBP is not solely a mechanical disorder but rather a complex inflammatory process. The pathogenesis of CLBP has been associated with pro-inflammatory cytokines, notably interleukin-6 (IL-6), which contributes to tissue damage and pain sensitization [5,6]. The consistent observation of elevated IL-6 levels in patients with chronic pain conditions indicates that it may serve as a potential therapeutic target [7].

De Queiroz et al. (2016) conducted a study examining the relationships between plasma inflammatory cytokines and pain and disability, revealing that TNF-α, sTNFR1, and IL-6 showed positive correlations with pain severity or intensity [8]. Extensive research on LBP and associated inflammation highlights the ongoing nature of symptoms, as assessed by severity; serum protein and mRNA levels of IL-6, IL-8, and TNF-α were notably higher in those with significantly elevated LBP [9,10,11]. IL-6 showed a positive association with somatic dysfunction, assessed by the Oswestry Disability Index (ODI) [10].

Vitamin D insufficiency is widely recognized as a contributing factor to musculoskeletal discomfort and CLBP [11]. Vitamin D possesses immunomodulatory properties and is crucial for regulating inflammatory responses. Research indicates that vitamin D supplementation can reduce pro-inflammatory cytokines, such as IL-6, and improve pain outcomes in various chronic pain conditions [12,13]. Multiple mechanisms explain how vitamin D impacts the risk of LBP, such as by regulating anti- and pro-inflammatory cytokines that affect pain and inflammation [14] and modulating pain by influencing sensory neuron excitability [15,16]. Moreover, an inverse correlation exists between inflammatory markers and serum levels of 25-hydroxyvitamin D (25(OH)D), a standard indicator of vitamin D status [17], with studies demonstrating decreases in inflammatory markers subsequent to vitamin D supplementation [18]. This deficiency-specific rationale matters biologically: vitamin D’s immunomodulatory and neuromodulatory actions are expected to yield clinical benefit only when a true deficit is present and can be corrected, since VDR-mediated suppression of pro-inflammatory cytokines requires substrate repletion. Supplementation in vitamin D-replete individuals would not be expected to produce comparable effects [19,20]. Accordingly, the present trial specifically enrolled patients with laboratory-confirmed vitamin D deficiency or insufficiency, as this population is central to the biological rationale and the interpretation of the study findings. Given the growing interest in vitamin D supplementation as an adjunctive treatment for LBP [21], a clearer understanding of the relationship between vitamin D status and LBP remains essential.

A widely used electrotherapeutic technique for the physiotherapeutic treatment of CLBP is interferential current (IFC) therapy, which applies two medium-frequency alternating currents that intersect within tissues to produce a low-frequency, amplitude-modulated current, enabling deeper tissue penetration with minimal discomfort [22]. Its analgesic effects are attributed to modulation of the autonomic nervous system, suppression of nociceptive transmission via the gate control mechanism, and stimulation of endogenous opioid pathways [23]. A randomized controlled trial further showed that IFC at 4 kHz modulated at 100 Hz had significant immediate analgesic effects, supporting its integration into multimodal physiotherapy programs for CLBP [24]. Evidence from a systematic review with meta-analysis confirms that IFC probably reduces pain intensity and disability post-treatment compared with placebo in chronic nonspecific LBP [25].

Exercise therapy is a cornerstone of evidence-based physiotherapy for CLBP, with robust evidence supporting its efficacy in reducing pain intensity, improving functional disability, and modulating the neuroinflammatory environment. A network meta-analysis identified strength training, Pilates, and core-based exercises as the most effective modalities for pain and disability management in CLBP [26], while meta-analytic evidence further confirms that stabilization exercises produce the strongest effects on pain and disability with treatment durations of 8 to 12 weeks [27]. Beyond structural benefits, exercise therapy reduces circulating pro-inflammatory cytokines, including IL-6 and TNF-α, addressing the symptomatic and biological dimensions of CLBP [28]. The combination of IFC therapy and core exercise has demonstrated greater reductions in pain and disability than either intervention alone [29], supporting an integrated multimodal approach to CLBP rehabilitation. However, evidence regarding vitamin D supplementation for LBP is mixed: while some trials report an analgesic benefit of correcting deficiency [20,30], systematic reviews pooling trials conducted in populations not selected for deficiency found no significant benefit of vitamin D monotherapy over placebo [19,31]. The evidence gaps testing vitamin D specifically as an adjunct to physiotherapy in a deficiency-confirmed population motivated the design of the present trial. Recent combined-modality trials, such as a 2025 randomized control trial (RCT) of motor control exercise plus transcutaneous electrical nerve stimulation in obese CLBP patients [32], similarly demonstrate that adjunctive interventions can outperform single-modality physiotherapy alone. No prior trial, to our knowledge, has tested vitamin D supplementation specifically as an adjunct to combined IFC-plus-exercise physiotherapy in a population selected for confirmed deficiency while also tracking a mechanistic inflammatory biomarker (IL-6) alongside clinical outcomes; this combination is the specific contribution of the present study.

The interplay between systemic inflammation, vitamin D status, pain sensitization, and functional disability in CLBP suggests that a combined therapeutic approach is beneficial. This approach integrates physiotherapy, including interferential current therapy and exercise therapy, alongside vitamin D supplementation. Specifically, we hypothesized that physiotherapy combined with vitamin D supplementation would produce greater improvements in pain and disability and a greater reduction in IL-6 than physiotherapy alone in CLBP patients with confirmed vitamin D deficiency or insufficiency. The primary outcomes were pain (NPRS) and disability (ODI) at six weeks. IL-6 and serum vitamin D changes were secondary, mechanistic outcomes. Therefore, this study aimed to assess the combined effects of physiotherapy and vitamin D supplementation on pain, disability, IL-6 expression, and vitamin D levels in CLBP patients.

## 2. Materials and Methods

### 2.1. Study Design and Setting

A two-arm, parallel-group, randomized controlled trial was conducted in accordance with the ethical principles outlined in the Declaration of Helsinki. The study received approval from the Local Ethics Committee at the University of Tabuk in Saudi Arabia (Approval no. UT-133-01-2021). All participants provided written informed consent. The research was carried out in King Fahd Specialist Hospital, Tabuk, Saudi Arabia. The study was registered at ClinicalTrials.gov (ID number: NCT07439874) and reported in accordance with the Consolidated Standards of Reporting Trials (CONSORT) guidelines [33] (Supplementary File S1).

### 2.2. Participant Recruitment

Screening for eligibility was performed by a senior orthopedician in diagnosing and managing CLBP conditions. Participants were recruited between 15 August 2024 and 15 January 2026. All participants underwent a comprehensive baseline evaluation that included demographic information, metabolic indicators (fasting blood glucose, HbA1c, lipid profile comprising total cholesterol, low-density lipoprotein [LDL], high-density lipoprotein [HDL], and triglycerides), an inflammatory biomarker (serum IL-6 levels in pg/mL), and vitamin D status (serum 25-hydroxyvitamin D [25(OH)D] levels in ng/mL). CLBP patients were divided into two groups (*n* = 30 per group). One group received physiotherapy alone, while the other group received physiotherapy and 60,000 IU of vitamin D3 tablets weekly for six weeks [12]. All patients participated in physiotherapy sessions for six weeks. Low back pain disability was assessed at baseline and after six weeks using the Arabic version of the ODI, with pain levels measured by the Numeric Pain Rating Scale (NPRS), serum IL-6 level, and vitamin D status.

### 2.3. Inclusion and Exclusion Criteria

Inclusion criteria:
  - CLBP persists for more than 3 months;
  - Male or female, aged 20–70 years;
  - Pain intensity of 4 or greater on the NPRS;
  - Able to understand and follow instructions;
  - Laboratory-confirmed vitamin D deficiency or insufficiency (serum 25(OH)D < 20 ng/mL) at screening.
Exclusion criteria:
  - Skeletal deformities;
  - Rheumatologic conditions;
  - Progressive motor weakness;
  - Incontinence;
  - History of cancer;
  - Use of systemic medications;
  - Systemic illnesses;
  - Fractures.

### 2.4. Sample Size Calculation

The sample size was calculated to achieve 80% power at a two-sided significance level of *α* = 0.05, aiming for a minimal clinically relevant difference of 2.0 points on the NPRS, using a comparison-of-means method [34]. This calculation assumed a pooled standard deviation for the NPRS based on prior literature/pilot estimates, yielding a base requirement of approximately 26 participants per group; this was inflated to 30 per group to account for an anticipated attrition rate of up to 15%. The calculation was performed using G*Power 3.1. The study included 60 participants, with 30 in each group.

### 2.5. Blinding and Allocation Concealment

**Figure 1:**
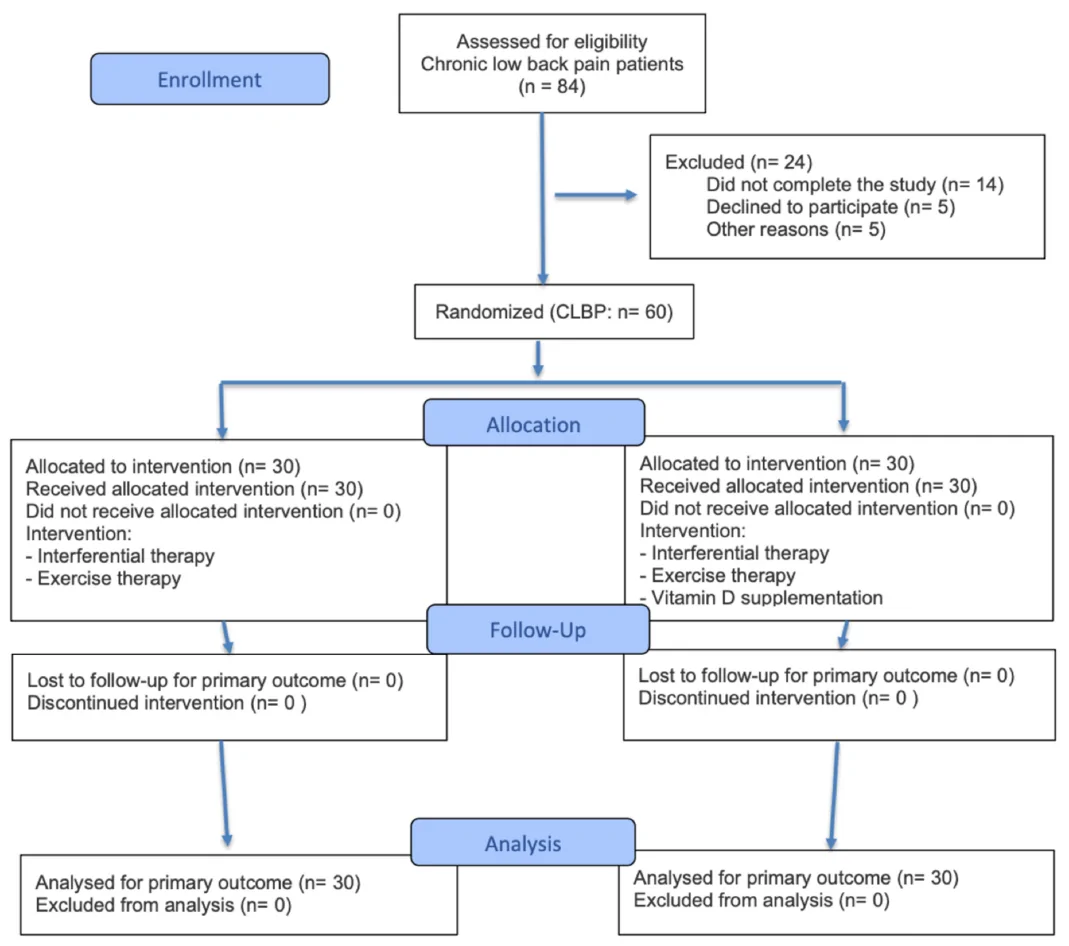
CONSORT flow diagram. Flow diagram of participants, including enrollment, allocation, follow-up (6 weeks), and analysis.

All participants were referred from the King Fahd Specialist Hospital, Tabuk, Saudi Arabia, for treatment and were provided with a pamphlet detailing the study’s procedures, benefits, and risks. A physiotherapist from the outpatient clinic explained the study protocol to the participants and invited them to take part. A total of 84 patients were assessed for eligibility, of whom 24 were excluded prior to randomization: 14 did not complete the pre-randomization screening/baseline assessment procedures, 5 declined to participate, and 5 were excluded for other reasons. After signing the informed consent and before the baseline assessment, participants were randomly assigned to one of two groups. The randomization sequence was computer-generated (simple randomization, 1:1 allocation ratio) by an expert independent of recruitment, treatment delivery, and outcome assessment. Enrollment (consent and eligibility confirmation) was performed by the recruiting physiotherapist, while allocation was performed by a separate physiotherapist not involved in recruitment or assessment. Group A (*n* = 30) received physiotherapy including IFC and exercise therapy, and Group B (*n* = 30) received physiotherapy in conjunction with vitamin D supplementation. A separate physiotherapist managed group allocation using an on-site computer system to ensure concealment; this system functioned as a restricted-access, sequentially concealed tool that released each assignment only after eligibility confirmation was finalized, without displaying the upcoming sequence, preventing foreknowledge of forthcoming allocations. Both groups received their respective therapies twice weekly for 6 weeks. A blinded physiotherapist evaluated NPRS and ODI, while a laboratory technician, blinded to group allocation, measured inflammatory biomarkers, including IL-6, and vitamin D levels at baseline and after the 6-week intervention period. Participants and treating therapists could not be blinded to allocation because Group B alone received a visible oral supplement, and no placebo capsule was administered to Group A. The CONSORT flowchart [30] is presented in Figure 1, and the CONSORT reporting checklist is attached to the manuscript (Supplementary File S1).

### 2.6. Outcome Measures

#### 2.6.1. Numeric Pain Rating Scale

The NPRS is a well-validated, unidimensional tool for measuring pain intensity. Patients assess their pain on an 11-point scale from 0 (no pain) to 10 (worst pain imaginable). The NPRS has demonstrated excellent test–retest reliability and construct validity in populations with LBP [34,35]. A 2-point change or a 30% drop from the baseline is usually considered the minimal clinically important difference (MCID) [36]. This tool effectively detects changes following physiotherapy interventions and provides a simple, quick way to monitor pain levels during treatment.

#### 2.6.2. Arabic Version of the Oswestry Disability Index Questionnaire

The ODI is a patient-reported (self-administered) disability questionnaire used to assess physical disability associated with back pain [37]. It has established internal consistency and test–retest reliability, along with concurrent validity against other disability measures in Arabic-speaking LBP populations [38,39]. A 10-percentage-point change is generally considered the MCID.

#### 2.6.3. Inflammatory Biomarkers: Interleukin-6 (IL-6)

IL-6 is a pro-inflammatory cytokine that is significantly elevated in individuals with CLBP, particularly in those with intervertebral disc degeneration [4]. Research indicates that serum IL-6 levels correlate positively with pain severity (*r* = 0.45–0.62) and disability scores among LBP patients [30]. This cytokine plays a crucial role in disc degeneration and nerve sensitivity, affecting disc structural deterioration and pain perception [3]. Typically, serum IL-6 levels range from 0 to 5 pg/mL; however, patients with CLBP often exhibit levels ranging from 10 to 30 pg/mL or higher [40].

The ELISA kit used (Calbiotech Inc., El Cajon, CA, USA) has manufacturer-reported intra-assay and inter-assay coefficients of variation of <10%, supporting analytical reliability.

#### 2.6.4. Vitamin D Parameters

Serum 25-hydroxyvitamin D [25(OH)D] is considered the most reliable marker for assessing vitamin D status [31]. Vitamin D levels can be categorized into three groups: deficiency (<20 ng/mL or <50 nmol/L), insufficiency (20–30 ng/mL or 50–75 nmol/L), and sufficiency (>30 ng/mL or >75 nmol/L) [41]. A significant number of patients with CLBP exhibit vitamin D deficiency or insufficiency, with studies showing that 60–85% have levels below the reference range [42,43]. Furthermore, low vitamin D levels are associated with increased pain severity, higher disability scores, and poorer treatment outcomes in individuals with CLBP [12].

### 2.7. Physiotherapy Intervention for CLBP

#### 2.7.1. Interferential Current Therapy

**Figure 2:**
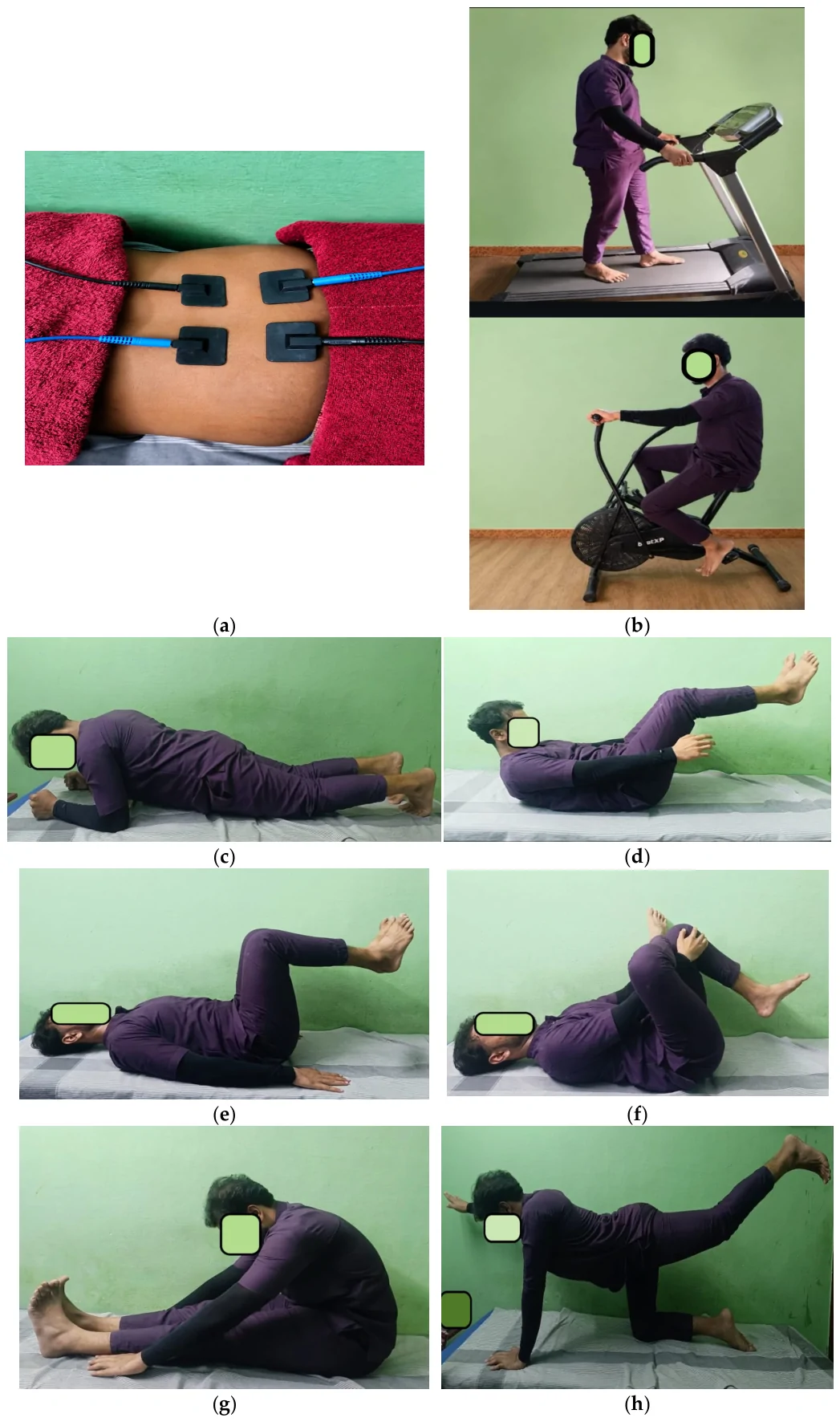
(**a**) Interferential current therapy for low back pain. (**b**) Treadmill and static bicycling. (**c**) Core strengthening—front bridge. (**d**) Core strengthening—pilates crunch. (**e**) Core strengthening—tabletop. (**f**) Gluteal stretch. (**g**) Hamstring stretch. (**h**) Bird-dog exercise.

Participants were instructed to relax their muscles during IFC, which required them to lie in a prone position. Four rectangular electrodes, each measuring 90 × 50 mm, were positioned at the levels of the L3 and L5 spines, with a spacing of 5 cm between them. These electrodes, designed for broader tissue coverage, utilized a quadripolar placement with an appropriate interferential mode to complete the pain circuit at the primary pain site. The current from each channel passed through the painful area. In the first channel, one electrode was placed to the right of L3, while the other was positioned to the left of L5. In the second channel, one electrode was placed to the left of L3, and the other was positioned to the right of L5. The carrier frequency of 4 kHz was modulated at 100 Hz and adjusted based on patient tolerance and the delivery mode (e.g., quadripolar sweep versus bipolar/pre-modulated). The skin was disinfected with rubbing alcohol, and flexible carbon-impregnated silicone rubber electrodes were applied using electroconductive gel and secured with tape to ensure optimal electrical contact. No patients were harmed during the treatment. A calibrated device (Sonopuls 692^®^; Enraf-Nonius BV, Rotterdam, The Netherlands) was utilized for 30 min to deliver the IFT [44]. Figure 2a explains the method of interferential current therapy.

#### 2.7.2. Exercise Therapy

The exercise therapy was designed to improve trunk muscle endurance and stability and was delivered by physiotherapists with more than 5 years of clinical experience through supervised sessions. To enhance reproducibility, the intervention is described below according to the FITT (Frequency, Intensity, Time, and Type) framework.

Frequency: Twice weekly, for six weeks (12 supervised sessions in total); no unsupervised home exercise component was included.

Intensity: Individualized; patients began each exercise at the difficulty level allowing completion of the minimum number of repetitions, progressing to the next level after completing the maximum repetitions for most sets during two consecutive visits.

Time: One-hour sessions comprising a 5-min light aerobic warm-up (stationary bike, elliptical trainer, or treadmill), the core-strengthening circuit, six static stretches (hamstring, gluteal, and lumbar muscles, held for three deep breaths, performed once each) before and after the circuit, and a cool-down.

Type: Supervised trunk/core-stabilization exercises, including prone and supine bridging, bird-dog, pelvic tilts, and partial trunk curls for 2–3 sets of 15–30 repetitions per exercise, adjusting the patient’s center of gravity when feasible. The protocol was adopted based on these studies [45,46]. Figure 2b–h show the exercises prescribed to the participants.

### 2.8. Sample Collection

Approximately 3 mL of peripheral blood was collected from each participant in EDTA (ethylenediaminetetraacetic acid) and plain vials. Serum was isolated, and all samples were stored at −20 °C until further processing. The baseline characteristics of 60 CLBP patients with vitamin D deficiency or insufficiency (<20 ng/mL) are depicted in Table 1.

### 2.9. Estimation of Serum Interleukin-6 Level by ELISA

Following the collection of blood samples in either plain or serum separator tubes, they were left undisturbed for ten to fifteen minutes at room temperature. By centrifuging the samples for ten minutes at 3500 rpm, the serum was separated. After careful transfer to different tubes, the serum was promptly refrigerated at −20 °C. Serum IL-6 levels were measured using a human interleukin-6 enzyme-linked immunosorbent assay (ELISA) kit (Calbiotech Inc., El Cajon, CA, USA). The average absorbance values for each set were determined after standards and samples were run in duplicate. The standard curve was used to plot OD values against standard concentrations of IL-6 to determine the serum IL-6 concentrations in each sample. The final IL-6 concentrations in each study sample were calculated by multiplying the corresponding dilution factors employed during sample preparation.

### 2.10. Serum Vitamin D Level and Supplementation

After blood samples were collected in plain or serum separator tubes, the tubes were left undisturbed at room temperature for 10–15 min. For serum separation, samples were centrifuged at 3500 rpm for 10 min. The serum was promptly stored at −20 °C after careful transfer to other tubes. Serum vitamin D levels were assessed using a human vitamin D ELISA kit (Calbiotech Inc., El Cajon, CA, USA). The average absorbance values for each set were determined after the standards and samples were run in duplicate. A standard curve was generated by plotting OD values against known vitamin D standard concentrations and was subsequently used to determine the vitamin D concentration of each sample. The final vitamin D concentration in each research sample was calculated by multiplying the respective dilution factors employed during the sample preparation. This weekly high-dose (60,000 IU) oral cholecalciferol regimen is consistent with repletion regimens recommended for vitamin D-deficient adults by the Endocrine Society Clinical Practice Guideline, which supports high-dose weekly or equivalent daily dosing to correct deficiency over 6–8 weeks [36]. Vitamin D3 was administered as an oral tablet, self-administered by the participant once weekly under the physiotherapist’s supervision during the twice-weekly clinic visits; adherence was monitored via a weekly pill count/diary check performed by study staff, with any missed dose recorded. Vitamin D supplementation corrects deficiency and may provide therapeutic benefits beyond simple repletion through pleiotropic mechanisms [47].

## 3. Statistical Analysis

Data were analyzed using SPSS version 26.0 (IBM Corp., Armonk, NY, USA). Normality was assessed with the Shapiro–Wilk test. Values are presented as *M* ± *SD*. No missing data were identified. Continuous variables (age, BMI, NPRS, ODI, fasting glucose, HbA1c, lipid profile, IL-6, and vitamin D) were compared between groups using independent samples *t*-tests; gender, the only categorical variable, was compared using the chi-square test. Three sets of comparisons were performed: (i) between-group comparisons of baseline variables (Table 1); (ii) within-group change from baseline to 6 weeks for each outcome, evaluated with paired *t*-tests (Table 2); and (iii) between-group comparisons of the magnitude of change for NPRS, ODI, IL-6, and vitamin D, evaluated with independent samples *t*-tests and reported with 95% CIs. A *p*-value of less than 0.05 was considered statistically significant.

## 4. Results

Participant flow and baseline characteristics: 60 CLBP patients with vitamin D deficiency or insufficiency (<20 ng/mL) were recruited and randomly assigned to two groups (*n* = 30 per group).

### 4.1. Baseline Characteristics of Intervention Groups

Sixty CLBP patients with vitamin D deficiency were randomized to Group A (physiotherapy only, *n* = 30) or Group B (physiotherapy plus vitamin D supplementation, *n* = 30). Baseline characteristics of the two groups are depicted in Table 1.

Table 1 presents the baseline demographic and clinical characteristics of participants in Group A (*n* = 30) and Group B (*n* = 30), confirming comparability between the two groups prior to intervention. There were no statistically significant differences between groups in age (37.91 ± 7.12 vs. 36.82 ± 8.13 years, *p* = 0.582), gender distribution (21/09 vs. 19/11, *p* = 0.062), or BMI (28.5 ± 2.15 vs. 27.9 ± 1.9 kg/m^2^, *p* = 0.257). Similarly, baseline pain and disability scores were comparable, with no significant differences in NPRS (5.17 ± 0.86 vs. 5.33 ± 0.79, *p* = 0.456) or ODI (27.43 ± 6.66% vs. 26.07 ± 4.49%, *p* = 0.357). Metabolic parameters, including fasting glucose (115.19 ± 22.63 vs. 118.90 ± 20.30 mg/dL, *p* = 0.506), HbA1c (6.50 ± 1.80% vs. 6.10 ± 1.13%, *p* = 0.306), total cholesterol (210.0 ± 30.66 vs. 214.60 ± 30.11 mg/dL, *p* = 0.559), LDL (121.10 ± 26.13 vs. 119.90 ± 27.14 mg/dL, *p* = 0.862), HDL (45.12 ± 6.21 vs. 44.50 ± 5.90 mg/dL, *p* = 0.693), and triglycerides (192.20 ± 56.71 vs. 190.66 ± 54.75 mg/dL, *p* = 0.915), also showed no significant intergroup differences. Likewise, baseline IL-6 (26.58 ± 6.80 vs. 27.11 ± 5.95 pg/mL, *p* = 0.876) and vitamin D levels (18.68 ± 2.58 vs. 19.68 ± 2.65 ng/mL, *p* = 0.144) were statistically similar between groups. These findings indicate that the two groups were well-matched at baseline, minimizing the likelihood that any post-intervention differences were attributable to pre-existing group disparities.

### 4.2. Outcome Measurements

**Figure 3:**
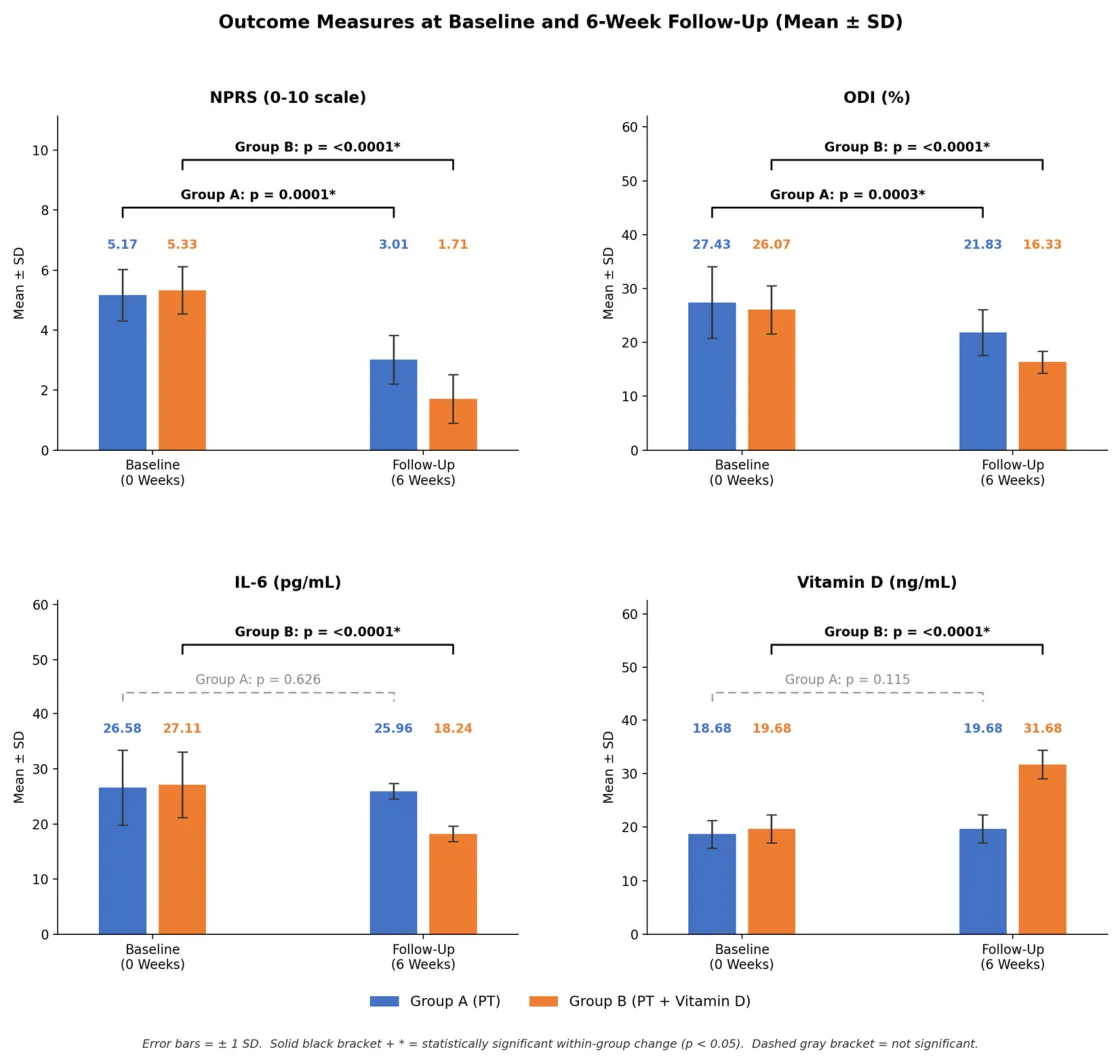
Bars represent group mean values, with error bars denoting ±1 standard deviation (*SD*). Brackets connect each group’s baseline and follow-up values; solid black brackets with an asterisk (*) denote a statistically significant within-group change (*p* < 0.05, paired *t*-test), and dashed grey brackets denote a non-significant change. Exact *p*-values are labeled above each bracket.

Table 2 and Figure 3 present the within-group and between-group changes in clinical and biochemical outcomes measured at baseline (0 weeks) and following six weeks of intervention in both treatment groups.

Pain and Disability Outcomes (NPRS and ODI): Both groups demonstrated statistically significant improvements in pain intensity and functional disability over the 6-week intervention period. In Group A (PT alone), NPRS scores decreased from 5.17 ± 0.86 to 3.01 ± 0.81, a mean reduction of 2.160 points (95% CI: (−1.72 to −2.59), *p* = 0.0001), while ODI scores fell from 27.43 ± 6.66% to 21.83 ± 4.25%, a reduction of 5.600% (95% CI: (−2.71 to 8.48), *p* = 0.0003). In Group B (PT + Vitamin D), the improvements were more pronounced: NPRS scores dropped from 5.33 ± 0.79 to 1.71 ± 0.81, a decrease of 3.620 points (95% CI: (−3.20 to 4.03), *p* < 0.0001), and ODI scores decreased from 26.07 ± 4.49% to 16.33 ± 2.05%, a reduction of 9.740% (95% CI: (−7.93 to 11.54), *p* < 0.0001). These findings indicate that while both interventions led to significant reductions in pain and disability, the addition of vitamin D supplementation to physical therapy resulted in substantially greater improvements in NPRS and ODI scores compared with physical therapy alone.

Inflammatory Marker and Vitamin D Status (IL-6 and Vitamin D): In Group A, IL-6 levels showed a negligible and statistically non-significant change, decreasing slightly from 26.58 ± 6.80 pg/mL to 25.96 ± 1.39 pg/mL (difference: 0.620, 95% CI: 3 (1.91 to 3.15), *p* = 0.626), and serum vitamin D levels increased marginally from 18.68 ± 2.58 ng/mL to 19.68 ± 2.65 ng/mL (difference: 1.00, 95% CI: (−0.251 to 2.25), *p* = 0.115), which was also not statistically significant. In contrast, Group B exhibited highly significant changes in both parameters: IL-6 levels declined substantially from 27.11 ± 5.95 pg/mL to 18.24 ± 1.39 pg/mL, a reduction of 8.870 pg/mL (95% CI: (−6.63 to 11.10), *p* < 0.0001), while vitamin D levels rose markedly from 19.68 ± 2.65 ng/mL to 31.68 ± 2.65 ng/mL, an increase of 12.00 ng/mL (95% CI: (10.63 to 13.36), *p* < 0.0001). These results suggest that vitamin D supplementation, when combined with physical therapy, effectively reduced systemic inflammation, as reflected by IL-6 levels, and successfully corrected vitamin D insufficiency, an effect not observed with physical therapy alone.

## 5. Discussion

This trial evaluated whether adding vitamin D supplementation to physiotherapy improves pain, disability, IL-6 levels, and vitamin D status in CLBP patients with confirmed vitamin D deficiency. Group B (physiotherapy plus vitamin D) showed significantly greater improvement than Group A (physiotherapy alone) across all four outcomes, as detailed in Section 4.2. The following sections interpret these findings in the context of the demographic, metabolic, and biomarker profile of the cohort and the existing literature.

### 5.1. Demographic and Metabolic Profile

The CLBP cohort (*n* = 60) was well matched for age and gender, minimizing potential confounding effects on clinical outcomes. However, individuals with group CLBP A and B demonstrated a significantly elevated BMI, corroborating research that identifies overweight and obesity as risk factors for the onset and persistence of CLBP [48]. The elevated BMI in individuals with CLBP may stem from increased mechanical stress on the spine, the release of pro-inflammatory cytokines from adipose tissue, and the sequestration of vitamin D in fat, which exacerbates deficiency [49]. Additionally, both groups demonstrated fasting glucose and HbA1c values that were numerically elevated relative to standard reference ranges, with evidence of dyslipidemia (increased total cholesterol and triglycerides). These values may be consistent with, but do not establish, impaired glucose regulation or metabolic syndrome, since formal diagnostic criteria (American Diabetes Association, 2024; the harmonized metabolic syndrome definition of Alberti et al., 2009) were not systematically applied in this study [50,51]. The elevated IL-6 levels further support the existence of a pro-inflammatory state in CLBP, consistent with findings linking neuroinflammation and peripheral sensitization to chronic pain [52].

In both groups, mean serum vitamin D was 18.5 ng/mL, below the 20 ng/mL threshold for deficiency, consistent with prior reports that 80–96% of CLBP patients exhibit deficiency [53,54]. The association between low vitamin D levels and increased pain is well documented, with studies reporting negative correlations between serum 25(OH)D levels and pain intensity [20,55]. Vitamin D likely provides analgesic effects by modulating cytokine production, regulating neuromuscular function through calcium, and interacting with vitamin D receptors (VDRs) on neurons in the dorsal root ganglion to diminish central pain sensitization [13].

### 5.2. Baseline Comparability

The two groups were comparable at baseline for age, gender, NPRS, ODI, IL-6, and vitamin D levels, confirming equivalent pain burden and inflammatory status before intervention. The relationship between Body Mass Index (BMI), CLBP, and diabetes mellitus is a complex interaction of mechanical, metabolic, and inflammatory pathways. Given the epidemic rise of metabolic disorders and obesity worldwide, it is imperative to understand the pathological link of BMI elevation as a bridge between diabetes and musculoskeletal degeneration for clinical management. Frontera WR, DeLisa JA (2010) reported that an elevated Body Mass Index—specifically when categorizing an individual as overweight (BMI ≥ 25.0 kg/m^2^) or obese (BMI ≥ 30.0 kg/m^2^)—significantly alters the physical forces acting upon the lumbar vertebrae [56]. Both groups had elevated BMI at presentation (BMI ≥ 25.0 kg/m^2^), with no significant difference between them. A notable BMI disparity between groups represents a potential confounding variable, as elevated BMI compounds vitamin D deficiency by sequestering vitamin D in adipose tissue and promoting adipose-mediated inflammation [48]. Future studies should stratify by BMI to evaluate its moderating effect on treatment response.

### 5.3. Pain and Functional Disability

The present study demonstrated that both PT alone and PT combined with vitamin D supplementation produced statistically significant improvements in pain intensity (NPRS) and functional disability (ODI) after six weeks; however, the combined intervention yielded substantially superior results across both primary outcomes. These findings are broadly consistent with established evidence supporting PT as an effective first-line intervention for CLBP.

Group B demonstrated significantly greater pain reduction than Group A, consistent with findings [12,38] reporting superior outcomes with combined vitamin D and physiotherapy for musculoskeletal pain. The analgesic benefit is attributable to vitamin D’s downregulation of pro-inflammatory cytokines (IL-6, TNF-α), upregulation of anti-inflammatory mediators, and interaction with nociceptive neurons’ VDRs via NGF and GDNF pathways [5,9]. These neuromodulatory effects, combined with physiotherapy’s biomechanical benefits, likely produced the greater improvement with the combined intervention pain reduction in Group B. Notably, however, not all systematic reviews have confirmed this effect. A meta-analysis by Zadro JR et al. [19] and a subsequent updated meta-analysis [31] reported that vitamin D supplementation alone was not superior to placebo for LBP, suggesting that its benefit may be contingent upon its combination with active rehabilitation and the presence of confirmed deficiency. This distinction likely explains the divergence between our positive combined-intervention finding and those null monotherapy results, since neither of those meta-analyses selected specifically for baseline vitamin D deficiency or tested vitamin D as an adjunct to active rehabilitation.

Functional disability, as assessed by the ODI, improved significantly in both groups after six weeks. However, Group B experienced much greater functional recovery compared with Group A, with a statistically significant difference between the groups. The ODI is a validated, widely used patient-reported (self-administered) measure of CLBP, known for high test–retest reliability and responsiveness to change after treatment [38]. A 10-percentage-point reduction is considered the minimal clinically important difference (MCID) for ODI [39]. Group B achieved an average reduction of about 9.7 percentage points, close to the MCID, while Group A’s decrease was only 5.6 percentage points, below the clinical significance threshold. This difference in functional recovery likely stems from the complementary effects of the combined intervention. Physiotherapy, involving structured exercises and manual techniques, addresses biomechanical issues, muscle deconditioning, and movement dysfunction common in CLBP [56,57]. Meanwhile, vitamin D supplementation promotes muscle protein synthesis, improves neuromuscular coordination, and mitigates inflammation-related muscle weakness [58]. The combination of these mechanisms in Group B probably explains the greater functional improvements seen. These results align with a 2024 systematic review of physiotherapy approaches for low back pain [58], which found that combining exercise therapy with adjunct interventions yields better outcomes than single treatments. The results are further consistent with a 2025 RCT combining motor control exercise with TENS in obese CLBP patients [32], although that trial’s obese-specific cohort, its use of TENS rather than IFC, and its lack of an inflammatory biomarker outcome limit direct comparability with the present study. In addition, functional disability in CLBP is shaped by psychosocial factors beyond the biochemical and biomechanical measures captured in the present trial, supporting our recommendation that future studies incorporate broader biopsychosocial assessment [59]. The randomized controlled trial by Yıldız, Canlı, Kocaman, & Alkan (2024) [60], which showed that adding Kinesio taping to conventional treatment improved outcomes in CLBP beyond conventional treatment alone, further supports evidence that adjunctive interventions combined with conventional physiotherapy consistently outperform physiotherapy alone in CLBP, paralleling the pattern observed with vitamin D supplementation in the present study.

### 5.4. IL-6 and Vitamin D Levels

The IL-6 and vitamin D levels in this study revealed the most striking contrast between the two groups. In Group A, neither serum IL-6 nor vitamin D levels changed significantly over the six-week intervention period (*p* = 0.147 and *p* = 0.136, respectively). This null result should not be read as confirming that PT alone has no effect on systemic inflammatory status: given the modest sample size and six-week duration, the trial was not powered to detect a smaller true effect on these biomarker outcomes. This finding is consistent with the broader understanding that exercise-based rehabilitation, in the absence of nutritional correction, does not reliably resolve the biological drivers of inflammation in vitamin D-deficient patients with CLBP [61,62].

In contrast, Group B demonstrated a large and highly significant reduction in IL-6 (18.24 pg/mL), alongside a 31.68 ng/mL rise in serum vitamin D, elevating levels from a deficient to a sufficient range. The marked IL-6 reduction in Group B is particularly significant, as IL-6 is a key pro-inflammatory cytokine implicated in the pathogenesis of CLBP, contributing to sensitization of nociceptors, promotion of pain chronification, and degradation of intervertebral disc and paraspinal muscle tissue [62,63]. Several studies have documented elevated IL-6 levels in patients with CLBP and an inverse relationship between vitamin D status and IL-6 concentrations [64]. Specifically, research has confirmed that hypovitaminosis D and CLBP may be mediated through the IL-6 pathway, with vitamin D receptors (VDRs) present on immune cells modulating cytokine production, including IL-6 suppression [65].

The immunomodulatory mechanism of vitamin D is well established: active vitamin D (1,25-dihydroxyvitamin D3) binds to VDRs present on macrophages, dendritic cells, and T lymphocytes, downregulating the transcription of pro-inflammatory cytokines, including IL-6 and TNF-alpha, while upregulating anti-inflammatory mediators [65,66]. A 2024 review by Fenercioglu et al. reinforced this mechanistic framework [64], and a 2025 systematic review confirmed that reduced vitamin D levels are independently associated with elevated IL-6 and TNF-alpha in musculoskeletal conditions [65]. A more recent systematic review similarly describes heterogeneous associations between serum vitamin D and chronic musculoskeletal pain across studies [67], underscoring that this relationship is not uniformly established across all populations. These converging findings offer a plausible explanation for why Group B achieved superior outcomes. One plausible, though statistically untested, explanation is that vitamin D supplementation reduced systemic inflammation via the IL-6 pathway, which may have potentiated the analgesic and rehabilitative effects of physiotherapy. However, no formal mediation analysis was performed, and only a single inflammatory biomarker (IL-6) was measured, so this pathway should be regarded as a hypothesis for future research rather than a demonstrated mechanism.

Although a recent meta-analysis by Lee et al. (2024) reported limited efficacy of vitamin D supplementation alone for CLBP, finding no significant pain reduction compared with control groups across ten RCTs (SMD: −0.130; 95% CI: −0.260 to 0.000), these findings do not contradict the present results [31]. Rather, they underscore an important distinction: vitamin D supplementation as a standalone intervention in populations not selected for deficiency may have limited benefit, whereas its administration as an adjunct to structured PT in vitamin D-deficient patients with CLBP yields a meaningful, clinically significant improvement in the combined intervention effect. The present study’s population presented with baseline deficiency, which may explain why supplementation exerted such a pronounced anti-inflammatory and pain-modulating effect. This interpretation is consistent with the existing literature suggesting that patients with 25-hydroxyvitamin D levels below 20 ng/mL are most likely to benefit from supplementation [66,67].

### 5.5. Limitations

The present study has several limitations that should be considered when interpreting the findings. First, the 6-week intervention period limits the ability to determine the long-term sustainability of the observed effects. Second, the inflammatory assessment was limited to IL-6, and no formal mediation analysis was conducted to examine whether changes in IL-6 mediated improvements in pain and disability. Therefore, the proposed IL-6-mediated mechanism discussed in Section 5.4 should be regarded as a biologically plausible hypothesis rather than a demonstrated causal pathway. In addition, the absence of a vitamin D supplementation-only group prevents isolation of the independent effect of supplementation from that of physiotherapy and precludes a formal statistical evaluation of whether the combined intervention produced a synergistic effect.

Furthermore, Group A did not receive a placebo capsule, and neither participants nor treating therapists were blinded to treatment allocation, as only Group B received the oral vitamin D supplement. Consequently, nonspecific expectation or performance effects cannot be excluded, particularly for the subjective outcomes of pain and disability (NPRS and ODI). Although the sample size was adequately powered for the primary pain outcome, it may have been insufficient to detect smaller between-group differences in the biomarker outcomes. Moreover, the trial was conducted at a single center, which may limit the generalizability of the findings to other populations and healthcare settings. Adherence to vitamin D supplementation was monitored using weekly pill counts and participant diaries rather than a more objective measure, such as interim serum vitamin D assessment. Finally, the external validity of the findings may also be influenced by the population-specific metabolic profile of the cohort, including the high prevalence of glycemic dysregulation and dyslipidemia, as well as the baseline imbalance in BMI between the intervention groups.

### 5.6. Recommendations

The findings of this study have several clinical and research implications. Routine screening for vitamin D status may be considered as part of standard assessment protocols for patients with CLBP. In individuals with a confirmed deficiency, correction of vitamin D levels may be considered as a potential adjunct to physiotherapy-based rehabilitation. Future research should stratify participants according to BMI, include a broader panel of inflammatory biomarkers, and extend the follow-up period to evaluate the long-term sustainability of treatment effects. In addition, optimal dosing regimens for vitamin D supplementation in this population should be investigated through larger randomized controlled trials. Finally, future studies employing a four-arm design, including physiotherapy alone, vitamin D supplementation alone, a combined intervention, and a placebo group, would allow clearer delineation of the independent and synergistic effects of these interventions.

### 5.7. Strengths and Practical Implications

This trial has several methodological strengths: a randomized, CONSORT-compliant design with blinded outcome assessment; a population selected for confirmed vitamin D deficiency rather than an unselected CLBP population (a common limitation of prior negative monotherapy trials); and the inclusion of a mechanistic biomarker (IL-6) alongside clinical outcomes. Practically, these findings support incorporating vitamin D screening into CLBP assessment where feasible and, in patients with confirmed deficiency, considering supplementation as a low-cost, low-risk adjunct to standard physiotherapy rather than a substitute for it.

## 6. Conclusions

This study demonstrates that combining physiotherapy with vitamin D supplementation is more effective than physiotherapy alone in reducing pain, reducing functional disability, and increasing serum vitamin D levels in CLBP patients over six weeks. The demographic and metabolic profile of the CLBP groups, characterized by BMI, numerically elevated glycemic and lipid markers, and markedly reduced vitamin D levels, highlights the systemic complexity of this condition and emphasizes the importance of comprehensive biochemical screening in CLBP management. While both treatments reduced pain and improved function, adding vitamin D provided a significant clinical benefit in NPRS and ODI scores that physiotherapy alone could not achieve within the same period. Overall, these results support considering routine vitamin D screening and supplementation for vitamin D-deficient CLBP patients as a valuable adjunct to physiotherapy-based rehabilitation, pending confirmation in larger, blinded, placebo-controlled trials.

## Figures and Tables

**Table 1:** Baseline characteristics of intervention groups.

Parameter	Group A (*n* = 30)	Group B (*n* = 30)			
	*Mean* ± *SD*	*Mean* ± *SD*	Dif.	(95% CI)	*p*-Value
Age (years)	37.91 ± 7.12	36.82 ± 8.13	1.090	(−5.04 to 2.86)	0.582
Gender (M/F)	21/09	19/11			0.062
BMI (kg/m^2^)	28.5 ± 2.15	27.9 ± 1.9	0.600	(−1.64 to 0.44)	0.257
NPRS	5.17 ± 0.86	5.33 ± 0.79	0.160	(0.26 to 0.58)	0.456
ODI (%)	27.43 ± 6.66	26.07 ± 4.49	1.360	(−4.30 to 1.57)	0.357
Fasting Glucose (mg/dL)	115.19 ± 22.63	118.90 ± 20.30	3.710	(−7.40 to 14.82)	0.506
HbA1c (%)	6.50 ± 1.80	6.10 ± 1.13	0.400	(−1.18 to 0.37)	0.306
Total Cholesterol (mg/dL)	210.0 ± 30.66	214.60 ± 30.11	4.60	(−11.10 to 20.30)	0.559
LDL Cholesterol (mg/dL)	121.10 ± 26.13	119.90 ± 27.14	1.200	(−14.96 to 12.56)	0.8.62
HDL Cholesterol (mg/dL)	45.12 ± 6.21	44.50 ± 5.90	0.620	(−3.75 to 2.51)	0.693
Triglycerides (mg/dL)	192.20 ± 56.71	190.66 ± 54.75	1.540	(−30.35 to 27.27)	0.915
IL-6 (pg/mL)	26.58 ± 6.80	27.11 ± 5.95	0.530	(−2.77 to 3.83)	0.876
Vitamin D (ng/mL)	18.68 ± 2.58	19.68 ± 2.65	1.0	(−0.35 to 2.35)	0.144

Values are expressed as *M* ± *SD*. Gender is expressed as frequency (M/F). An independent samples *t*-test was used for continuous variables and a chi-square test for categorical variables. *p* < 0.05 was considered statistically significant. NPRS = Numeric Pain Rating Scale; ODI = Oswestry Disability Index; BMI = Body Mass Index; IL-6 = interleukin-6; CI = confidence interval. Groups A and B did not differ significantly at baseline for any demographic, clinical, or biochemical variable (all *p* > 0.05; Table 1), confirming successful randomization.

**Table 2:** Outcome measurements at baseline and follow-up.

Outcomes	Baseline (0 Weeks) *M* ± *SD*	Follow-Up (6 Weeks) *M* ± *SD*	Difference	95% CI	*p*-Value
Group A (PT)					
NPRS	5.17 ± 0.86	3.01 ± 0.81	−2.160	(−2.59 to −1.72)	0.0001 *
ODI (%)	27.43 ± 6.66	21.83 ± 4.25	5.600	(−2.71 to 8.48)	0.0003 *
IL-6 (pg/mL)	26.58 ± 6.80	25.96 ± 1.39	0.620	(1.91 to 3.15)	0.626
Vitamin D (ng/mL)	18.68 ± 2.58	19.68 ± 2.65	1.00	(−0.251 to 2.25)	0.115
Group B (PT + Vitamin D)					
NPRS	5.33 ± 0.79	1.71 ± 0.81	3.620	(−3.20 to 4.03)	<0.0001 *
ODI (%)	26.07 ± 4.49	16.33 ± 2.05	9.740	(−7.93 to 11.54)	<0.0001 *
IL-6 (pg/mL)	27.11 ± 5.95	18.24 ± 1.39	8.870	(−6.63 to 11.10)	<0.0001 *
Vitamin D ng/mL)	19.68 ± 2.65	31.68 ± 2.65	12.00	(10.63 to 13.36)	<0.0001 *

Values are expressed as *M* ± *SD*. Paired samples *t*-test was used to compare baseline and follow-up values within each group. * Statistically significant at *p* < 0.05. PT = physiotherapy; NPRS = Numeric Pain Rating Scale; ODI = Oswestry Disability Index; IL-6 = interleukin-6; CI = confidence interval.

## Data Availability

All data associated with the research study are presented in the manuscript.

## Supplementary Materials

The following supporting information can be downloaded at https://www.mdpi.com/article/10.3390/life16081288/s1, File S1: CONSORT reporting checklist.
